# Economic microbiology: exploring microbes as agents in economic systems

**DOI:** 10.3389/fmicb.2024.1305148

**Published:** 2024-02-21

**Authors:** Nicola Luigi Bragazzi, Woldegebriel Assefa Woldegerima, Anna Siri

**Affiliations:** ^1^Laboratory for Industrial and Applied Mathematics (LIAM), Department of Mathematics and Statistics, York University, Toronto, ON, Canada; ^2^Department of Health Sciences (DISSAL), Postgraduate School of Public Health, University of Genoa, Genoa, Italy; ^3^United Nations Educational, Scientific and Cultural Organization (UNESCO) Chair, Health Anthropology Biosphere and Healing Systems, University of Genoa, Genoa, Italy; ^4^Department of Wellbeing, Nutrition and Sport, Pegaso University, Naples, Italy

**Keywords:** economic biology, microbiology, microbiodiversity, biological markets, mathematical modeling

## Abstract

Microbial communities exhibit striking parallels with economic markets, resembling intricate ecosystems where microorganisms engage in resource exchange akin to human market transactions. This dynamic network of resource swapping mirrors economic trade in human markets, with microbes specializing in metabolic functions much like businesses specializing in goods and services. Cooperation and competition are central dynamics in microbial communities, with alliances forming for mutual benefit and species vying for dominance, similar to businesses seeking market share. The human microbiome, comprising trillions of microorganisms within and on our bodies, is not only a marker of socioeconomic status but also a critical factor contributing to persistent health inequalities. Social and economic factors shape the composition of the gut microbiota, impacting healthcare access and quality of life. Moreover, these microbes exert indirect influence over human decisions by affecting neurotransmitter production, influencing mood, behavior, and choices related to diet and emotions. Human activities significantly impact microbial communities, from dietary choices and antibiotic use to environmental changes, disrupting these ecosystems. Beyond their natural roles, humans harness microbial communities for various applications, manipulating their interactions and resource exchanges to achieve specific goals in fields like medicine, agriculture, and environmental science. In conclusion, the concept of microbial communities as biological markets offers valuable insights into their intricate functioning and adaptability. It underscores the profound interplay between microbial ecosystems and human health and behavior, with far-reaching implications for multiple disciplines. To paraphrase Alfred Marshall, “the Mecca of the economist lies in economic microbiology.”

## Introduction

Economic decision-making is paramount to serving the goals of individuals, private enterprises, and societal organizations. In the former stance, decision-making serves private goals, whereas in the latter case, it serves public goals. In both cases, the aim is to attempt to control or, at least, reduce uncertainty and efficiently allocate and make use of limited, constrained resources (Galli and Battiloro, 2019).

Whilst classical and neoclassical theories have emphasized the rational aspect of economic decision-making processes (Virlics, 2013), contemporary bio- and neuro-behavioral models have been trying to incorporate bounded or restricted rationality, irrationality, inconsistency, time inconstancy, judgment, and cognitive biases in an increasingly realistic way to reflect a complex society that is facing more and more asymmetries, inequalities, crises, and shocks (Tversky and Kahneman, 1974).

For this purpose, these frameworks have leveraged information and knowledge from biology, physiology, and biochemistry/biophysics, as well as from psychology, and neuroscience, building bridges across disciplines that are usually conceived as unrelated and are siloed apart. This has enabled scholars to shed light on apparently unexpected or overlooked determinants of decision-making processes and drivers of economic choices (Livet, 2010). Also, this has allowed capturing real-life economic computations, which can be rather slow and error-prone, far from achieving *equilibria* in a reliable fashion, or staying stable, and frame-invariant, and are imperfectly implemented in real-world settings, which are constantly under flux, and exhibit high levels of uncertainty and noise (Barendregt et al., 2022).

According to the so-called “rational choice model,” the human, as *Homo oeconomicus*, is a completely, perfectly, consistently rational agent, narrowly self-focused and self-interested, who always maximizes utility/profit. This model has been gradually replaced by other models, including the “survival model,” borrowed from sociobiology (Magee, 1993), which aimed at explaining human behaviors as attempts to enhance economic fitness. These behaviors can be either cognitive or non-cognitive, rational, or instinctual, and are genetically, hormonally, and environmentally driven (Ludwig and Welch, 2019). *Homo oeconomicus* as a conceptual framework to model human activities and behaviors has been replaced by *Homo duplex*, the idea that human is a dynamical dual agent, consisting of a biological make-up shared with animal creatures and a distinctive rational part (Kluver et al., 2014).

The incorporation of evolutionary biology, and, more generally speaking, biology is known as bioeconomics, a term coined by the British biologist Reinheimer (Reinheimer, 1913), conceptualized by the Romanian statistician and economist Georgescu-Roegen (Georgescu-Roegen, 1971), and popularized by several prominent economists, including the American economists Rothschild (Rothschild, 1992) and Magee (1993). Within the arena of bioeconomics, some super specialties have emerged, including geno-economics (Navarro, 2009; Benjamin et al., 2012a,b) and neuro-economics (neuro-finance or neuro-marketing) (Glimcher and Fehr, 2014), even though they have spurred controversies about the soundness of the adopted methodology and the reproducibility of findings (Benjamin et al., 2012a).

However, even if the human body hosts a remarkably diverse community of microorganisms (called altogether as microbiota) (Gilbert et al., 2018), the role of microbes in economic decision-making has been relatively overlooked. Recent advancements in next-generation sequencing technologies, including metagenomics, have profoundly transformed our comprehension of the human microbiome and we now regard it as an intimately linked entity known as the meta-organism or holo-biont. Our exploration of the human microbiome has revealed its involvement in a more extensive array of biological processes functions, events, and diseases than previously envisioned, including cardiovascular diseases, malignancies, neuropsychiatric disorders, like autism, obesity, metabolic syndrome, and various others (Gomaa, 2020; Shi, 2023).

While our understanding of how the microbiome influences brain function and behavior remains incomplete, mounting evidence underscores a strong connection between the brain and the gut. In this context, several instances highlight the critical role of the microbiome in cross-kingdom communication and interactions. A number of comprehensive reviews of the most up-to-date literature (Berg and Sensen, 2017; Houdek, 2018) have showcased how the analysis of human microbiome data has ushered in a paradigm shift in our comprehension of its contributions not only to disease and physio-pathology but also, and especially, to physiology and decision-making.

Houdek (2018) has summarized studies exploring the impact of microorganisms and chemosignals on human economic behavior, discussing the biological roots of economic development, and emphasizing how infectious agents, in particular, parasitic ones and sexually transmitted organisms, and microbiota (referred to as “human holobiont”) can influence human decision-making and behavior in areas like risk-seeking, impulsivity, social dominance, empathy, political views, and gender differences. *Toxoplasma gondii*, responsible for toxoplasmosis, which is associated with a variety of neuropsychiatric and behavioral conditions, is used as a case study to shed light on the influences of micro-organisms on economic preferences, personality characteristics, and human appearance. Finally, chemosignals are shown to affect decision-making, especially in the domain of social preferences, which suggests that integrating microbiology and economics can lead to predictions for economic sciences, based on the emerging paradigm of economic holobiont.

However, this review focuses on physio-pathological aspects. Collectively, evidence from multiple research *foci* points out the key role played by our microbiome, which must be recognized as an indispensable partner in economic behaviors and the processes of decision-making. A closer integration of microbiome research into the field of economics would enable us to unlock deeper insights into these intriguing connections.

Here, we offer the foundation of “economic microbiology,” defined as the science of the two-way interactions between the realm of microbes and the economic arena (Table 1).

**TABLE 1:** Comparative analysis of economic microbiology and market dynamics: exploring parallels in concepts and applications.

Concept in Economic Microbiology	Parallel in Economic Markets	Examples/Applications
Resource Exchange.	Trade and Barter Systems.	Microbial nutrient cycling, Market trade of goods.
Competition for Resources.	Market Competition.	Microbial survival strategies, Business strategies in competitive markets.
Mutual Benefit Alliances.	Business Partnerships.	Symbiotic relationships in microbes, Joint ventures in business.
Impact on Host Health.	Economic Impact on Society.	Influence of gut microbiota on human health, Economic policies’ impact on social welfare.
Microbial Diversity and Stability.	Market Diversity and Stability.	Diversity in microbial communities ensures ecosystem health, Diverse markets prevent economic collapse.
Influence of Environmental Factors.	Influence of Market Conditions.	Environmental changes affect microbial behavior, Market trends and policies affect business strategies.
Adaptation to Changing Environments.	Market Adaptation and Evolution.	Microbes adapting to new environments, Businesses evolving in response to market changes.
Microbial Communication (Quorum Sensing).	Market Communication and Signaling.	Communication among bacteria for collective decision-making, Market signals influencing investor decisions.
Resource Depletion and Sustainability.	Resource Management in Economics.	Overuse of resources leading to microbial habitat loss, Economic principles of sustainable resource use.
Influence of Social and Economic Factors on Microbiome.	Socioeconomic Influences on Market Trends.	Impact of lifestyle and diet on human microbiome, Socioeconomic factors shaping market demands.

## Microbial communities as biological markets and microbes as economic agents

The “Biological Market Theory” (BMT), formulated by Noë and Hammerstein (1994) and (Noë and Hammerstein, 1995), is a complex conceptual framework rooted in evolutionary biology and in the comparative study of animal behavior explored with economic lenses. Acknowledging (and incorporating) insights from evolutionary ecology which categorizes all the types of interactions that two different species can have as neutralism, competition, synergism or mutualism, predation or parasitism, commensalism, and amensalism, BMT posits that animals engage in various forms of cooperative and mutualistic behaviors, even when there is the potential for cheating or exploitation, because of the “comparative benefits” they receive from these interactions. The economic concept of “comparative advantage” suggests that mutually beneficial trade relationships can occur when two (or more) self-interested entities produce the same goods, provided they have differing efficiencies in producing those goods. According to this theory, when these entities engage in trade, they maximize their consumption by specializing in the production of the goods they can produce most efficiently. The less efficient entity specializes even more, and both entities benefit from the trade by consuming more goods than they could produce individually. Not only humans, but also animals can engage in “biological marketplaces” where they exchange commodities in the form of goods or services (such as food, grooming, protection, cooperative hunting, or mating opportunities) to increase their fitness and reproductive success. This theory explains why cooperative interactions among animals occur, which cannot be easily explained by other theoretical frameworks, including traditional natural selection or kin selection theories that often focus on competition and selfish behaviors. BMT shifts the focus to cooperative and mutually beneficial interactions: in biological markets, animals can be thought of as “traders” that can assess the value of different trading partners, make economic decisions, and engage in transactions by offering valuable resources to the most beneficial and reliable partners in exchange for something they need, in order to maximize their own fitness.

Biological Market Theory has proven effective in elucidating cooperative behaviors among numerous animal species. Microorganisms, too, engage in cooperative actions, both with their hosts and fellow microorganisms, which can be analyzed using economic concepts. However, the conventional approach does not usually involve applying market principles to scrutinize these interactions. In this context, Werner et al. (2014) have expanded the framework of BMT to examine its relevance to evolutionary biologists investigating microbes. The authors have explored several economic strategies that microbes employ to enhance their success within these biological markets. The authors have shown that adopting an economic market framework offers a valuable tool for generating precise and intriguing predictions regarding microbial interactions, which encompass aspects such as the development of partner discrimination, strategies related to resource accumulation, the choice between specialized and diversified mutualistic services, and the significance of spatial configurations like groups and *consortia*. There exists considerable potential for exploring the evolutionary dynamics of microbial systems, and the application of BMT can effectively organize and guide research in this area by characterizing the strategic investments made by microbes under diverse conditions.

A significant portion of Earth’s microbial life thrives within intricate communities where metabolic exchanges are crucial. Microbes engage in the trading of essential resources (a variety of metabolites including essential amino acids, sugars, fatty acids, and cofactors) to facilitate their own growth, akin to the way countries exchange goods within modern economic markets. Drawing inspiration from these parallels, Enyeart et al. (2015) have extended BMT and the theory of “comparative benefits/advantages” to the world of single-celled organisms by developing mathematical models of genetic circuits, which enable the exchange of a common resource, specifically signaling molecules essential for bacterial growth. Through these models, the authors have aimed to illustrate how comparative advantage interactions can manifest in microbial communities. In the first scenario, referred to as “Conception 1,” the authors have controlled production rates using external inducers, which allows the exploration of the specialization parameter space. In the second scenario, or “Conception 2,” the circuits self-regulate through feedback mechanisms. These models demonstrate that these genetic circuits can, indeed, exhibit comparative advantage dynamics. This form of cooperation is particularly advantageous under challenging external conditions and when the cost of production is not excessively high.

Tasoff et al. (2015) have further expanded BMT, by constructing a framework grounded in the principles of general equilibrium theory (GET) borrowed from economics, according to which markets and other economic systems dynamically evolve and interact in such a way that each agent independently optimizes their consumption, production, and exchange decisions, ensuring that there is no excess supply or scarcity of goods at the current price. This framework can be applied to the study of microbial communities engaged in resource exchange, enabling the forecasting of their population dynamics. This adapted, modified model, known as biotic GET (BGET), provides an *a priori* explanation of the production and allocation of metabolites and of the instantaneous growth rate advantages associated with microbial trade, based on the growth needs, metabolic capabilities, and intercellular transport rates of each microbial species, thus unveiling several insights that have relevance in understanding both microbial ecology and the engineering of synthetic communities. The economic concept of comparative advantage plays a pivotal role in facilitating mutualistic trade among microbes: More in detail, the BGET model indicates that microbial communities can proliferate more rapidly when individual species are incapable of independently generating essential resources, necessitating inter-species trade. These dynamics foster metabolic specialization and heighten intercellular resource exchange. Furthermore, findings highlight a fundamental tradeoff for species engaged in trade, one that involves balancing growth rate against relative population abundance. The specific environmental conditions, favoring either group selection or individual selection, influence the strategies that species adopt along this spectrum of growth and abundance. To empirically validate these findings, the authors have conducted experiments employing a synthetic *consortium* of *Escherichia coli* cells, confirming that the results align with the predictions of their proposed model (Tasoff et al., 2015).

Of note, adding to Tasoff et al.’s work (Tasoff et al., 2015), the principle of the importance of inter-species interactions, particularly in the context of resource exchange, for the proliferation of microbial communities can be applied to different microbial populations and settings. For instance, algae and cyanobacteria play distinct roles within this framework, especially when comparing ecosystems like the human gut to aquatic environments such as ponds or lakes. In the human gut, the microbial community is primarily composed of heterotrophs, organisms that obtain their energy by consuming organic substances produced by other organisms. These microbes rely on the breakdown of complex organic compounds ingested by the host, and they engage in a complex web of interactions. In contrast, aquatic ecosystems like ponds or lakes host a more diverse array of microbial life, including both phototrophs and heterotrophs. Algae and cyanobacteria are primary producers, capable of photosynthesis, and form the base of the aquatic food web, producing essential resources not just for themselves but for heterotrophic organisms as well. The presence of both producers/phototrophs and decomposers/heterotrophs creates a more self-sustaining ecosystem in that producers generate organic matter using sunlight, carbon dioxide, and water, which then serves as food for heterotrophs. Heterotrophs break down this organic matter, releasing nutrients back into the environment in a form that can be reused by producers, thus closing the nutrient cycle. This dynamic interplay between producers and decomposers in aquatic systems exemplifies the ecological principle of resource exchange and recycling, contributing to the overall productivity and stability of the ecosystem. In contrast, the gut microbiome’s reliance on a continual influx of nutrients from the host’s diet reflects a different kind of ecological balance, where microbial communities are adapted to extract and recycle nutrients from complex organic compounds provided by the host, and another type of economic system/organization.

In conclusion, conceptual frameworks borrowed from the economic arena can be successfully applied to the human microbiota, presenting novel, fresh perspectives and allowing the exploration of either natural or engineered microbial communities through the lenses of economic theories that have evolved over the past century (Pusa et al., 2019).

On the other hand, microbes generate essential metabolic resources, but a portion of these resources can escape into the surrounding environment (the so-called concept of “leaky microbial trade”). Other microbes can harness these “leaked” resources, adapt their metabolic production accordingly, and influence the resource pool available to all. Kallus et al. (2017) have delved into a model that explores the coevolution of metabolite concentrations, production regulation, and population frequencies. This scenario involves two types of cells producing two distinct metabolites. Within this model, the authors have uncovered three intriguing paradoxes where changes that should logically benefit a cell type end up causing harm. For instance, one paradox arises when a cell type becomes more efficient at metabolite production, yet its relative frequency in the population decreases—or conversely, the overall population growth rate declines. Another paradox manifests when a cell type manipulates its counterpart’s production to maximize its own instantaneous growth rate, only to achieve a lower final growth rate than if it had not intervened.

These paradoxes shed light on the intricate and (sometimes) counterintuitive dynamics that emerge within even the simplest microbial economies, showing that, similarly to human economies, also microbial ones are imperfect, with “free riders” lurking around the corner. Studying the imperfection of microbial trades can potentially deepen our understanding of human imperfect trades and markets.

Last, of note, Verstraete et al. (2007) have devised a theory called “Microbial Resource Management” (MRM), which focuses on deepening our comprehension of the latest advancements in microbiology and their applications in managing microbial resources, especially in open systems where microbial ecology is dynamic. This novel theoretical framework highlights the importance of understanding microbial communities and *consortia*, their interactions, and the management of microbial resources for environmental safety, health, renewable energy production, and sustainable environments, emphasizing a new mindset for microbial ecologists and environmental microbiologists, and considering various principles and theories in microbial communities. These include the principle outlined by the Italian economist Vilfredo Pareto in 1897, who was the first to describe how, in open competitive systems, a distribution pattern emerges where 20% of the population acquires 80% of resources. Conversely, the remaining 80% strives for greater efficiency, propelling overall performance improvement (Verstraete et al., 2022). This principle (known as the 80/20 rule, the law of the vital few, or the principle of factor sparsity) is applicable to microbial communities as well, being a useful concept for understanding resource sharing in stable microbial communities and guiding the understanding of the evolutionary trajectory of evolving communities. For instance, in soil ecosystems, examining the abundance of microbial *taxa* against their role in bioconversion often reveals a Pareto-like distribution, though the exact 80/20 ratio may not always be observed. This distribution pattern is also evident in engineered ecosystems like anaerobic digestion. Therefore, assessing microbial community ecology, vitality, and interaction effectiveness can be done through this metric, which offers more comprehensive insights than merely listing species presence or absence and emphasizes functional rather than taxonomic aspects of communities, suggesting a focus on transactional interactions among genotypes/ecotypes in a microbial market economy (Timmis et al., 2023).

The relevance of economic principles, like the Pareto principle, to explain phenomena and events such as competition and cooperation in microbial communities cannot be overstated, noting that competition may be more significant in unstable or evolving communities. Some important applications of the Pareto principle are related to contexts of microbial community dysbiosis and disease, suggesting that Pareto conformity might be associated with health and non-conformity with disease. A deviation from the typical Pareto distribution in microbial communities might be associated with dysbiosis, with this deviation indicating an imbalance in the microbial community, and potentially leading to health issues. In a healthy system, microbial communities often adhere to the Pareto principle, with a balanced distribution of resources among various microbes. However, in diseased states, this balance might be disrupted, leading to a disproportionate distribution of resources and an altered microbial composition, which could contribute to or signify disease. For example, in the field of infectious diseases, heterogeneity in transmission, which represents a challenge for infectious disease dynamics and control, can be modeled according to the 80/20 rule, which has been confirmed for a range of communicable diseases, ranging from influenza to malaria and the recent “Coronavirus Disease 2019” (COVID-19) outbreak (Woolhouse et al., 1997; Chen et al., 2015; Cooper et al., 2019; Liu and Zheng, 2023).

However, there is an urgent need for further exploration of the Pareto principle’s application in diverse microbial communities and various contexts, including health and disease status and environmental settings. Also, a fully formally developed theoretical framework comprehensively integrating economic concepts and translating them into the microbiological arena is still lacking.

The application of economic lenses is anticipated to significantly enhance our understanding of microorganisms and their role in the functioning of our planet and all macroscopic life. Despite recent scientific and (bio-) technological advancements, determining their activity and growth status *in situ* and *in vivo* remains a challenge, with the available body of research typically focusing on optimal growth conditions while studying microbial activity. However, the latter is linked to growth, which is influenced by nutrient availability in varying environmental conditions. As such, most research on microorganisms leaves a gap in understanding the behavior and function of microbes in slow-growing states, under growth-limiting conditions. A central aspect of economics is the concept of scarcity, which addresses the challenge of meeting unlimited human desires and needs with finite resources. This scarcity influences the monetary value assigned to goods and services and guides the resource allocation decisions of both governments and private entities. Scarcity is crucial for microorganisms as well, as they often encounter severe growth limitations in nature, which leads to the activation of unique pathways producing various secondary metabolites, and results in specific responses to slow growth, including survival strategies, gene expression, and cell differentiation, which are not fully understood (Gonzalez and Aranda, 2023).

## Microbes as markers of socioeconomic status and societal disparities

Moving from the microbial communities to the host, socioeconomic disparities in health and mortality are well-documented (Braveman et al., 2010), yet the biological mechanisms driving these connections remain less clear. Concurrently, growing attention is being paid to the gut microbiome as a potential influencer of human health, though our understanding of its broader environmental and social determinants remains limited.

For instance, Bowyer et al. (2019) have examined the relationship between gut microbiota composition and socioeconomic factors at both the individual and area levels, using a well-defined twin cohort as the study population, encompassing 1,672 healthy volunteers drawn from the TwinsUK twin registry, all of whom possessed data pertaining to at least one measure of socioeconomic status, as well as existing fecal 16S rRNA microbiota data, along with all relevant covariates. The findings revealed that the associations between gut microbiota composition and socioeconomic status remained robust even after accounting for known health factors linked to the microbiome. Conversely, adjusting for socioeconomic status partially mitigated the associations between health and the microbiome. The authors also observed that twins who differed in their Index of Multiple Deprivation scores exhibited significant distinctions in terms of microbiota composition dissimilarity. This suggests that the greater the disparity in the Index of Multiple Deprivation scores within twin pairs, the more pronounced the differences in their microbiota composition, pointing out that socioeconomic status might influence the makeup of the gut microbiota and its potential role as a mediator in the variations associated with socioeconomic status.

In the existing scholarly literature, this interplay has been leveraged to explain disparities in the colonization of multi-drug resistant bacterial strains (Zuniga-Chaves et al., 2023), incidence and mortality rates from chronic degenerative diseases (Miller et al., 2016; Virtanen et al., 2019; Ahn et al., 2023; Dixon et al., 2023), and related outcomes, showing that the persistence and widening of socioeconomic disparities in morbidity and mortality from degenerative disorders often share common pathogenic features, some of which involve changes in the composition, diversity, and functioning of the gut microbiota. Research has shown that socioeconomic status can account for from 11–12% up to 18–22% of the variability in diversity and richness of the colonic microbiota (Miller et al., 2016; Ahn et al., 2023).

## The (economic) interactions between humans and microbes−at the individual level

Animal models, human neuroimaging, and lesion studies have illuminated the impact of the gut microbiota on the interplay between the central and enteric nervous systems through the gut-brain axis (Gao et al., 2020; Liang et al., 2022). This influence extends to brain regions associated with fundamental emotional and cognitive processes, as well as with the insurgence of psychological issues and neurological diseases, including stress, autism, depression, Parkinson’s disease, and Alzheimer’s disease. However, the extent to which the gut microbiota influences decision-making in healthy humans has remained largely unexplored.

Some trials in animal and human models have shown that the administration of probiotic/prebiotic formulations can have psychotropic effects and, in individuals with neuropsychiatric diseases, can mitigate against the disorder, modulate neural and immunological pathways, and significantly enhance brain health (Messaoudi et al., 2011a,b; Kim et al., 2018; Suganya and Koo, 2020).

Recently, Dantas et al. (2022) have succeeded in establishing a functional connection between the gut microbiota and decision-making in healthy individuals, particularly in scenarios involving risk and time. To investigate this, the authors conducted a placebo-controlled, double-blinded study with two groups over two sessions separated by a 28-day interval. Participants were administered daily doses of either probiotics or a placebo. The authors aimed to determine whether sustained and controlled probiotic consumption would impact risk-taking behavior and choices related to the timing of rewards using incentivized economic tasks. Findings revealed a significant reduction in risk-taking behavior and an increase in choices oriented toward the future in the group receiving probiotics, as compared to the placebo group. These results provide compelling experimental evidence, for the first time, suggesting a potential functional role of the microbiota-gut-brain axis in decision-making. This opens up new avenues for potential clinical applications and enhances the understanding of the underlying neural mechanisms involved in risk-taking behavior and choices related to the timing of rewards.

## The (economic) interactions between humans and microbes−at the collective level

Moving from humans to societies, human activities significantly impact microbial communities and their pivotal role in delivering ecosystem services of significant importance, spanning from wastewater treatment to bioremediation, and the production of biofuels and pharmaceuticals. Anthropogenic pressure can disrupt what has been called microbial biodiversity or “microbiodiversity” (Han et al., 2023). All this can result in economic losses arising due to external effects stemming from the horizontal and vertical transfer of microbiodiversity between hosts and natural environments. Microbiodiversity, an integral component of biodiversity as a whole, holds a unique status as a natural resource within ecosystems, primarily due to the option values associated with the evolutionary potential of microbes, particularly when they are host-associated. Additionally, microbiodiversity offers insurance value in the face of changing environments.

Furthermore, institutions and organisms in terms of cultural and social norms play a crucial role in establishing policies aimed at managing and regulating the prevalence of circulating pathogens. This is not only a matter of global public health but also has significant economic implications, as vividly demonstrated during the COVID-19 pandemic. The widespread shutdowns and restrictions implemented to curb the spread of the virus resulted in substantial economic losses. The COVID-19 pandemic serves as a stark reminder of the intricate interplay between microbes and human societies. It underscores how the presence and transmission of pathogens can disrupt the functioning of economies and societies at large. Consequently, institutions are tasked with the challenging responsibility of striking a balance between safeguarding public health and minimizing the economic impact of pathogen-related measures. This requires a dynamic and adaptive approach that takes into account the evolving nature of microbial threats and their repercussions on human well-being and economic stability.

## State-of-the-art and future directions

Microbial communities have a fascinating resemblance to economic markets: they comprise a diverse array of microorganisms engaged in intricate interactions mimicking economic transactions in human markets. Within microbial communities, a fundamental aspect is the exchange of resources. Microbes collaborate and compete, swapping nutrients, metabolites, and signaling molecules, akin to the trading of commodities (goods and services) in human markets, creating a dynamic network of resource exchange. Just as businesses specialize in specific goods or services, microbial communities exhibit a division of labor. Different microbes within these communities often specialize in particular metabolic functions, contributing to the overall stability and efficiency of the ecosystem. Cooperation and competition are central dynamics in microbial communities. Microbes form alliances to secure resources or create advantageous conditions, mirroring companies forming partnerships for mutual benefit. Conversely, they engage in competition, with some species outcompeting others, much like businesses striving to dominate market share.

The human microbiome, composed of trillions of microorganisms residing within and on our bodies, is also a marker of socioeconomic status. Multiple social and economic factors interplay to shape the composition of the gut microbiota, resulting in persistent social inequalities that continue to hinder the achievement of universal healthcare access, directly impacting both the duration and quality of people’s lives. This lays the foundation for the development of future interventions aimed at alleviating the healthcare disparities associated with the interplay of biological, social, and economic factors.

Also, the human microbiome holds a significant influence on human health and decision-making. Microbes inhabiting the gut can influence the production of neurotransmitters, affecting mood and behavior. This, in turn, indirectly shapes human decisions, impacting choices related to diet, emotions, and more. Human activities have substantial repercussions on microbial communities. Factors ranging from the individual to the societal level, such as dietary choices, antibiotic usage, and environmental changes can disrupt these ecosystems. For instance, antibiotics can disturb microbial balance within the body, leading to imbalances, while changes in land use and pollution can alter microbial communities in natural environments. Beyond their natural roles, microbial communities are harnessed by humans for various applications, that often involve deliberate manipulation of microbial interactions and resource exchanges to achieve specific goals.

This broad overview has some limitations, in that it has provided examples of the application of economic theory to the functioning of microbial communities and populations, but the list of examples is far from being exhaustive. Future studies should explore, within the context of economic theory, the roles of further microbial processes, such as the dynamics of prophages and bacteriophages, quorum sensing, programmed cell death, the release of hydrolytic enzymes that contribute to common goods and the production of accessible organic carbon, as well as the behaviors of non-phototrophic carbon-fixing bacteria, biofilm formation, motility, and the mechanisms of sporulation and encystment, Additionally, given that microbial *consortia* have been evolving for 3 to 4 billion years, another (perhaps even more valuable and insightful) exercise could be systematically studying microbial behavior that might inform the development of a new, comprehensive theory of community energetics or economics, offering a deeper understanding of the economic behaviors of human societies.

Said so, the concept of microbial communities as biological markets offers valuable insights into their intricate functioning and adaptability, underscoring the intricate interplay between microbial ecosystems and human health and behavior, with far-reaching implications for fields such as medicine, agriculture, and environmental science. These microbial *consortia* represent what de Jonge et al. (2023) have conceptualized as the “metamicrobiome” and what Verstraete et al. (2007) have called the “metagenome of knowledge,” linking “the microbial communities on and inside our body,” integrating “the deep sea, the deep underground and the deep intestine” along the “environmental *continuum*” in and around us, and encompassing human health and well-being, environmental sustainability, and planetary health (Verstraete et al., 2007).

In conclusion, to paraphrase (Marshall, 2013), “the Mecca of the economist lies in economic microbiology.”

## Author contributions

NB: Conceptualization, Writing–original draft, Writing–review and editing. WW: Writing–review and editing. AS: Writing–review and editing.
